# Pelvic Morphology, Body Posture and Standing Balance Characteristics of Adolescent Able-Bodied and Idiopathic Scoliosis Girls

**DOI:** 10.1371/journal.pone.0070205

**Published:** 2013-07-17

**Authors:** Georgios A. Stylianides, Georges Dalleau, Mickaël Begon, Charles-Hilaire Rivard, Paul Allard

**Affiliations:** 1 Department of Kinesiology, Towson University, Towson, Maryland, United States of America; 2 CURAPS-DIMPS, Faculté des Sciences de l’Homme et de l’Environnement, Université de la Réunion, Le Tampon, France; 3 Department of Kinesiology, University of Montréal, Montreal, PQ, Canada; 4 Human Movement Laboratory, Research Centre, Saint-Justine Hospital, Montreal, QC, Canada; 5 Department of Orthopedic Surgery, Sainte-Justine Hospital, Montreal, PQ, Canada; University of South Australia, Australia

## Abstract

The purpose of this study was to determine how pelvic morphology, body posture, and standing balance variables of scoliotic girls differ from those of able-bodied girls, and to classify neuro-biomechanical variables in terms of a lower number of unobserved variables. Twenty-eight scoliotic and twenty-five non-scoliotic able-bodied girls participated in this study. 3D coordinates of ten anatomic body landmarks were used to describe pelvic morphology and trunk posture using a Flock of Birds system. Standing balance was measured using a force plate to identify the center of pressure (COP), and its anteroposterior (AP) and mediolateral (ML) displacements. A multivariate analysis of variance (MANOVA) was performed to determine differences between the two groups. A factor analysis was used to identify factors that best describe both groups. Statistical differences were identified between the groups for each of the parameter types. While spatial orientation of the pelvis was similar in both groups, five of the eight trunk postural variables of the scoliotic group were significantly different that the able-bodied group. Also, five out of the seven standing balance variables were higher in the scoliotic girls. Approximately 60% of the variation is supported by 4 factors that can be associated with a set of variables; standing balance variables (factor 1), body posture variables (factor 2), and pelvic morphology variables (factors 3 and 4). Pelvic distortion, body posture asymmetry, and standing imbalance are more pronounced in scoliotic girls, when compared to able-bodied girls. These findings may be beneficial when addressing balance and ankle proprioception exercises for the scoliotic population.

## Introduction

Scoliosis is characterized by a deformation of the spine and rib cage distortion [1]. The most frequent form is adolescent idiopathic scoliosis (AIS) which mostly affects young girls [2]. Most studies compare scoliotic girls to a group of able-bodied girls to identify abnormal pelvic growth [3], asymmetrical trunk posture [4–6] and standing imbalance [7,8]. Nevertheless, the relationship between these parameters is still unclear.

Attempts have been made to link standing balance to body posture [4,9], to morphology [10], and to curve type [11], or to associate scoliotic severity to standing imbalance [12]. These studies confirm that scoliosis and its progression are related to more than a single type of biomechanical factors. However, they do not address which variables are determinant and best characterize AIS girls per se.

Simoneau et al. [13] advocate a sensory integration hypothesis to explain balance control problems observed in AIS. This concept is based on the dynamic regulation of sensorimotor integration by the inappropriate weighting of sensory inputs. They conclude that AIS girls have difficulty in reweighing sensory inputs following a brief period of sensory deprivation. Bruyneel et al. [14] also attribute the variability in ground reaction forces during forward and lateral step initiation to the ontogenesis of adaptive strategies. In opposition, a biomechanical hypothesis gives importance to trunk shape and posture adjustments. This is supported by Stylianides et al. [3] who have observed a significant correlation between the Cobb angle and pelvic abnormal growth. Others like Burwell et al. [15], Goldberg et al. [16], and Ramirez et al. [17] found strong relationships between spinal deformity and asymmetrical postures reinforcing the biomechanical concept. The debate is still open since these studies compared the results of scoliotic to non-scoliotic girls and addressed only a single type of parameters; either motor control or biomechanical features characterizing AIS.

Multivariate statistical approaches facilitate the interpretation of data based on variance estimation. Factor analysis is a method to explain much of the variance in the data of a population with relatively few factors. The significant variables within a factor are often used to describe the concerned population [18]. These analyses were applied to gait analysis to reduce the number of variables and identify those with a greater explanatory interpretation [19,20]. To our knowledge this approach was never applied to describe and identify the main neuro-biomechanical characteristics in AIS.

Pelvic morphology [3], body posture [5] and standing balance [10] were studied in scoliotic and non-scoliotic, but never all of them in a single study. There is a biomechanical linkage between these parameters [4,15] but the mechanism is still unknown. If standing balance is a dominant factor in AIS, then exercise based therapy alone, or in combination with other orthopedic approaches, should be encouraged as an effective means of rehabilitation. The first objective of this study is to determine how pelvic morphology, body posture, and standing balance variables of scoliotic girls differ from those of a comparable able-bodied group. This will identify which variable is affected by scoliosis and by how much. To define which set of parameters best describes the scoliotic and able-bodied girls respectively, the second objective is to classify neuro-biomechanical variables in terms of a lower number of unobserved variables called factors (factor analysis), and best characterize both populations respectively.

## Materials and Methods

The full study was reviewed and approved by the ethics committee of Sainte-Justine hospital. Twenty-eight scoliotic girls participated in this study. All procedures were explained to each subject and a written informed consent was obtained from each subject and their parents or guardians prior to testing. The selection of the subjects was performed by an orthopedic surgeon using the definition given by Bunnell [21]. All had right thoracic curves with an average Cobb angle of 35.0° ± 7.6°. None of the scoliotic subjects was under active treatment. As shown in Table 1, their average age was 12.9 ± 1.6 years while their height and mass were 155.1 ± 9.9 cm and 44.7 ± 9.5 kg, respectively. The body mass index (BMI) was also calculated since body morphology has an effect on standing balance of able-bodied [22] and scoliotic girls [10]. The mean BMI was 18.4 ± 2.9 and it was within the normal range. Subjects wearing foot orthosis or showing any other signs of postural orthopedic or neurological complaints (including limb length discrepancies) were excluded from the study. The mean demographic characteristics of the untreated scoliotic group are also given in Table 1. The nonscoliotic or able-bodied group consisted of 25 girls who were examined by an orthopedic surgeon to ensure the absence of spinal deformity, foot problems (including limb length differences), back pain or any other signs of postural orthopedic or neurological disorders. No statistical difference was found between the groups in terms of age, height, mass and BMI.

**Table 1:** Mean values and standard deviations for age, height, mass, BMI and Cobb angle of the scoliotic and able-bodied groups and their *p* values.

Group	n	Age (years)	Height (cm)	Mass (kg)	BMI	Cobb angle (degrees)
AIS	28	12.9±1.6	155.1±9.9	44.7±9.5	18.4±2.9	35.0±7.2
Able-bodied	25	13.1±1.4	156.9±6.9	46.0±7.4	18.6±2.4	
*p* value		0.6302	0.4216	0.0564	0.8138	

Body posture orientation was determined with the subject standing barefoot with the heels spaced by 23 cm and the feet pointing externally by 15° [23]. The bony landmarks identified by Pasha et al. [24] were used to describe pelvic morphology and alignment. The landmarks included the right and left anterior (ASIS) and posterior (PSIS) superior iliac spines, as well as the first sacral (S1) vertebra. Trunk orientation was characterized by the anatomical reference points given by Nault et al. [4]. They correspond to the S1 and the seventh cervical (C7) spinous processes, the right and left acromions and the inferior angles of the scapula. With the subjects maintaining a quiet stance, the skin lying over the identified anatomical landmarks was lightly touched with the tip of the stylus of a Flock of Bird system (Ascension Technologies, Burlington, VT, USA) to record their 3D coordinates. This procedure was applied by Nault et al. [4] and Stylianides et al. [3] to describe pelvic morphology and trunk posture in girls. The linear and angular errors of this electromagnetic system is 0.76 mm and 0.1° respectively [25] and they are within the values reported for video-based systems where spherical markers of 10mm in diameter are used to monitor pelvic and trunk motions in standing [24].

All three-dimensional measurements were calculated with respect to S1 as the origin and positive values were along the right, anterior and upward directions. Three sets of variables were studied, namely, pelvic morphology, body posture, and standing balance. Pelvic morphology is represented by its mean right and left width obtained by averaging the distances of the ASIS and PSIS from S1 for each side respectively; the mean pelvic depth and height were calculated similarly. The pelvic orientation given by its sagittal tilt, frontal tilt and transverse rotation is calculated according to Pasha et al. [24], while trunk inclinations are calculated by the angle sustained by the line joining S1 to C7 and the vertical in the sagittal and frontal planes. Because the scoliotic deformity affects the whole trunk shape, two additional trunk orientations were calculated; lower and upper trunk. Lower trunk posture is defined by the angle between the vertical and the line joining S1 to scapular midpoint (S1-Sca) and that of the upper trunk is given by the vertical and the line defined by the scapular midpoint to C7 (Sca-C7). Transverse rotations were determined at the shoulder by the angle formed by the line joining the acromions and the transverse axis, and at the scapula by the angle given by the line joining their inferior angles also with respect to the transverse axis.

After standing balance was measured, the Flock of Bird instrumentation was removed so that it did not interfere with the following standing balance experiment [4]. Postural balance was measured with each subject standing on a force plate (AMTI OR6-5, Newton, MA) with the feet positioned as described above and arms parallel to the trunk. Subjects focused on a target placed at 1.2m ahead and located at eye level [10,26] for a period of 64 s [4,10] while data were collected at a frequency of 64 Hz. The center of pressure (COP) displacements along the anteroposterior (AP) and mediolateral (ML) directions were calculated from the unfiltered forces and moments [10]. For each of the three trials, the AP and ML COP range and speed were calculated and then averaged for each subject; no trials were rejected. The former is the difference between the maximal and minimal COP values given in a direction and is indicative of the ease of maintaining upright stance [13]. The COP speed is the sum of COP elementary displacements in a direction divided by the duration of the trial. It corresponds to the neuromuscular system demand [27] to avoid loss of balance. These conventional variables characterize the quality of standing balance along the AP and ML axes. Rotational sway about the vertical axis was proposed by Dalleau et al. [28] in the context of a global postural system control mechanism. It is assumed that the rotational or torsional movement of the trunk is under the control of the free moment (Tz) acting at the COP. This was supported by Beaulieu et al. [29] who reported an important contribution of the free moment for subjects standing on a single limb. This free moment represents the extent of an asymmetric control of the trunk about the vertical axis during standing. After normalizing it with respect to the body mass of the subject, its mean, range and RMS values were calculated for each trial and averaged for each subject.

To determine differences between the scoliotic and able-bodied groups, MANOVAs were performed on the pelvic morphology, body posture, and standing balance parameters. This was followed by planned comparisons to examine the specific effects when the MANOVAs reached a significant level. A Bonferroni correction procedure was applied to control Type 1 error by adjusting the *p* values in the analysis of the aforementioned variables [30]. A *p* value equal or less than 0.05 was considered as significant in all statistical analyses.

Factor analysis was carried out afterwards on all the variables to define which sets of variables or factors best describe the scoliotic and able-bodied groups respectively. It is used to explain much of the variance in the observed variable of a population with relatively few factors. The variables were classified into four factors where the relative contribution of each of the observed variables is given by a loading value. To facilitate interpretation, it is suggested [31] that each factor is labeled according to its statistically significant variables, namely those having a loading value of 0.7 or above [32].

## Results

**Figure 1:**
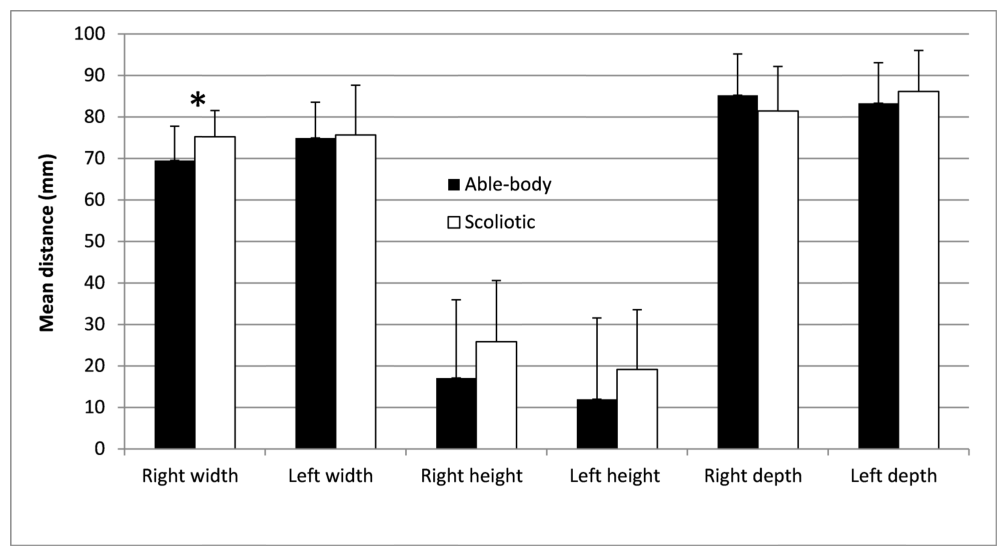
Mean and standard deviation of the pelvic morphology variables for the able-bodied and scoliotic groups. The * denotes a statistically significant difference with *p* < 0.05.

**Figure 2:**
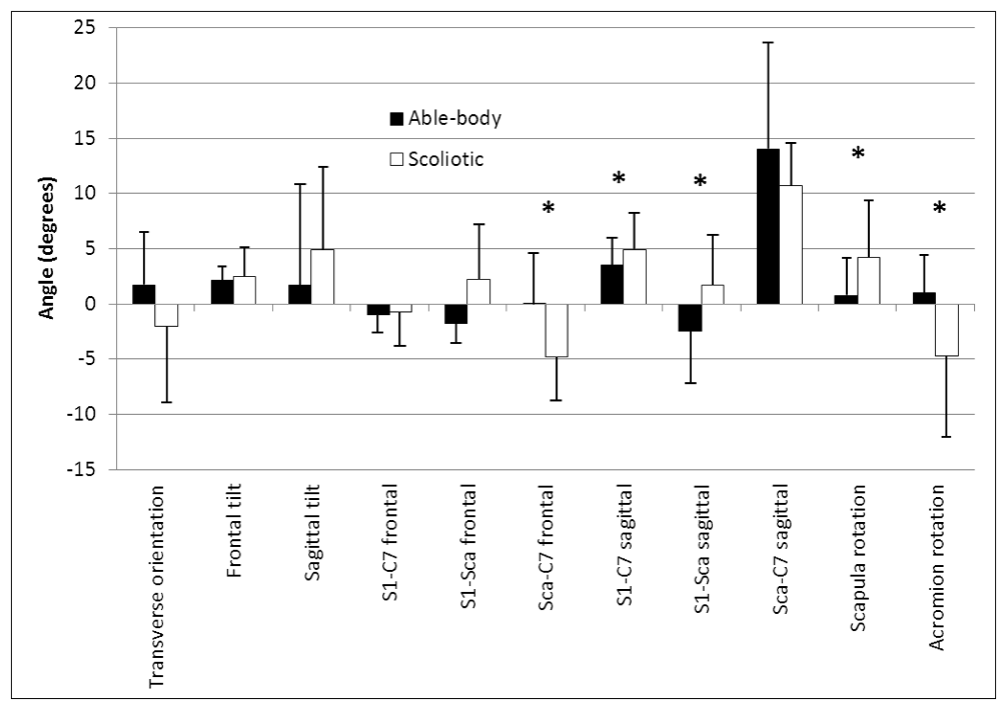
Mean and standard deviation of the pelvis and trunk orientation variables for the able-bodied and scoliotic groups. The * denotes a statistically significant difference with *p* < 0.05.

**Figure 3:**
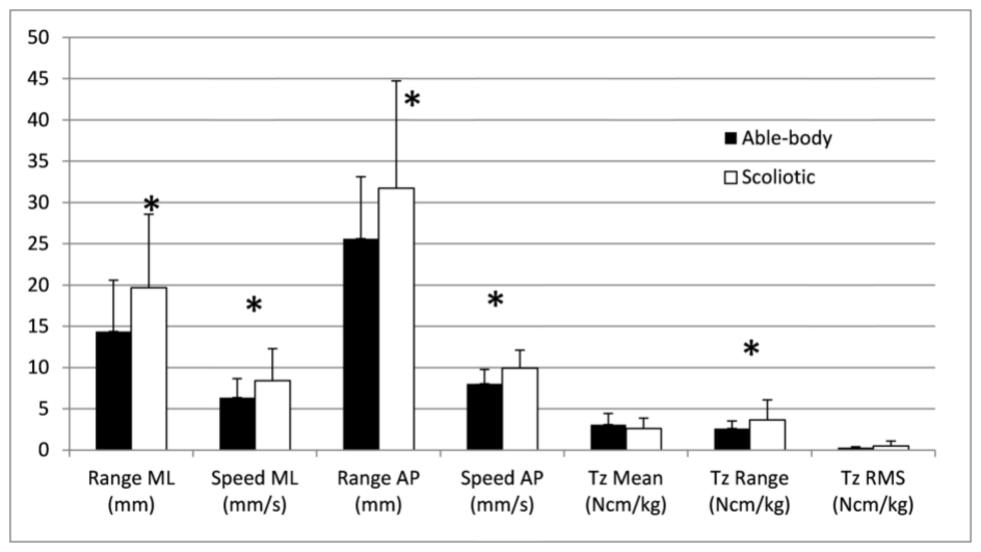
Mean and standard deviation of the standing balance variables for the able-bodied and scoliotic groups. The * denotes a statistically significant difference with *p* < 0.05.

The mean and standard deviations of the pelvic morphology, body posture and balance parameters for the able-bodied and scoliosis groups are given in Figures 1, 2, and 3. MANOVAs revealed a statistical difference between the groups (*p* < 0.001) for each type of parameters. Generally, the values were greater for the scoliotic group. The right pelvic width was statistically larger in the scoliotic group by 5.7 mm (*p* = 0.0061) as reported in Figure 1. The spatial orientation of the pelvis was similar for both groups with values approaching zero degrees; however, five of the eight trunk postural variables of the scoliotic group given in Figure 2 were significantly different from those of the able-bodied girls. Similarly, five values of the seven standing balance variables were statistically higher for the scoliotic girls, as shown in Figure 3.

A factor analysis revealed that nearly 60% of the variation is explained by 4 factors as shown in Table 2. The able-bodied and scoliotic girls are characterized first by Factor 1, then by the others since their respective eigenvalues decrease with the number of factors. Variables with a loading value of 0.7 and above are statistically significant and used to categorize or describe each factor. For both groups, each factor can be associated to a set of variables. Able-bodied girls are best described by standing balance variables (Factor 1), then with those associated to body posture (Factor 2) and pelvic morphology (Factors 3 and 4). The scoliotic girls are best described by the pelvic variables (Factor 1) followed by body posture (Factor 2), pelvic variables again (Factor 3) and lastly by standing balance variables (Factor 4). BMI did not appear in any of the factors for either group.

**Table 2:** Eigenvalues and cumulative Eigenvalues expressed in percentage, as well as the factors 1 to 4 and their loading factor values for the able-bodied and scoliotic groups. Statistically significant values equal or above 0.7 are in bold characters.

	Able-bodied				Scoliosis			
Factor	1	2	3	4	1	2	3	4
Eigenvalues	6.02	5.13	2.79	2.25	4.91	3.41	3.27	2.88
Cumulative (%)	24.08	44.60	55.77	64.77	19.62	33.27	46.34	57.86
BMI	-0.06	-0.51	0.25	0.39	0.01	-0.31	-0.57	0.16
Pelvic width right (mm)	0.03	0.30	-0.21	**0.72**	0.33	0.23	0.42	0.01
Pelvic width left (mm)	0.19	0.67	0.10	-0.08	-0.35	0.31	**0.74**	-0.17
Pelvic height right (mm)	-0.20	0.03	**0.89**	0.11	**0.89**	-0.24	-0.05	-0.01
Pelvic height left (mm)	-0.19	0.07	**0.93**	0.06	**0.95**	0.15	-0.03	0.14
Pelvic depth right (mm)	-0.23	-0.46	0.28	0.60	0.10	-0.12	**-0.78**	0.11
Pelvic depth left (mm)	0.01	0.03	0.27	**0.89**	0.22	-0.40	0.29	0.09
Pelvic transverse orientation	-0.32	-0.59	-0.08	-0.26	-0.09	0.18	**-0.86**	0.01
Pelvic frontal tilt	-0.06	-0.59	-0.48	0.07	-0.16	**-0.79**	0.12	-0.13
Pelvic sagittal tilt	-0.08	0.12	**0.90**	0.07	**0.94**	0.04	-0.01	0.01
S1-C7 frontal (degree)	-0.24	**0.81**	-0.04	0.08	-0.08	**0.79**	0.16	0.06
S1-Sca frontal (degree)	-0.47	0.60	0.08	0.26	-0.05	**0.90**	0.12	-0.13
Sca-C7 frontal (degree)	-0.03	0.66	0.06	-0.30	-0.21	-0.21	0.07	0.29
S1-C7 sagittal (degree)	0.13	0.63	0.30	-0.13	0.01	0.01	0.01	-0.43
S1-Sca sagittal (degree)	-0.25	**0.75**	0.26	0.07	-0.21	0.28	0.10	-0.47
Sca-C7 sagittal (degree)	0.28	0.40	0.41	-0.47	0.40	-0.43	-0.22	0.14
Scapula rotation (degree)	-0.65	-0.35	0.28	0.00	0.05	0.65	-0.51	-0.09
Acromion rotation (degree)	-0.65	0.07	0.32	0.00	0.02	0.31	-0.67	0.22
Range ML (mm)	**0.88**	0.02	-0.10	-0.06	0.04	0.09	0.16	**-0.92**
Speed ML (mm/s)	**0.80**	0.05	-0.25	-0.24	-0.05	0.05	0.13	**-0.82**
Range AP (mm)	0.22	0.23	-0.26	-0.25	-0.17	-0.13	0.01	-0.66
Speed AP (mm/s)	**0.71**	0.36	-0.12	-0.47	-0.17	-0.01	0.18	**-0.79**
Tz Mean (Ncm/kg)	0.09	-0.19	0.15	-0.22	-0.20	-0.39	-0.52	0.03
Tz Range (Ncm/kg)	**0.88**	-0.16	0.04	0.10	-0.03	-0.07	-0.30	**-0.70**
Tz RMS (Ncm/kg)	**0.82**	-0.34	-0.10	0.14	0.25	0.07	0.07	-0.16

## Discussion

It is difficult to have a unified description of pelvic morphology, body posture, and standing balance parameters of AIS girls from the literature because studies often focus on a single type of parameters at a time. Furthermore, different scoliotic subjects have participated in the various studies; the Cobb angle varies considerably; more than a single curve type are included or girls with different forms of scoliosis are selected. For example, it has been well documented that AIS girls display standing imbalance [7,11], have asymmetric body postures [5,6] and abnormal pelvic geometry [3,15]. However, only a few reported associations between standing balance and curve type [11], body morphology [10], and body posture [9] within the same group of scoliotic subjects. The present study supports these observations but also adds to these results on pelvic morphology abnormalities. The latter is an important factor related to the Cobb angle progression [3] and can involve the lumbar curve [33]. Though Stylianides et al. [3] reported pelvic morphology in both able-bodied and scoliotic girls and that sagittal spinopelvic balance was analyzed in non-scoliotic [34] and AIS subjects [35], no one has related it to body posture and standing balance yet. Our results have shown in a single group of scoliotic girls differences relative to able-bodied girls in pelvic geometry, body posture, and standing balance.

In this study, the factor analysis revealed four factors that explained about 60% of the variance in each group. Within each factor, variables with a loading factor of 0.7 or more were used to identify the significant ones and to label each factor. This approach has never been done before in the analysis of biomechanical data in AIS. However, multivariate analysis was used by Sadeghi et al. [36] to determine the contributions of the lower-limb in gait of people without impairments and by Astephen et al. [37] to explore the gait and neuromuscular pattern changes in the osteoarthritic knee.

Able-bodied girls adopt a relatively symmetric posture [4] and have little pelvic geometry abnormalities [3,24,34]. This explains in part why these characteristics are found in the lesser three factors. Whereas the values of these variables do not vary much within the able-bodied girls population, balance variables were reported to be dependent of body morphology [22] and gender [38]. It appears that standing balance is sensitive to body conditions in non-scoliotic individuals and that could explain in part its predominant contribution in the first factor.

In scoliotic girls, it is surprising that balance is present in the fourth factor. This implies that imbalance is an important factor, but secondary to abnormal pelvic morphology and distorted body posture identified in the first three factors. Simoneau et al. [13] suggest a sensory integration hypothesis where the impairments in the dynamic regulation of sensorimotor integration in AIS are due to the inappropriate weighting of sensory inputs. Our results do not question this assumption but rather relate it to a biomechanical hypothesis which is dependent of pelvic morphology and body posture. Pelvic geometry comes out both in the first and third factors in the scoliotic group. Stylianides et al. [3] have shown that asymmetrical bone growth is significantly correlated with the Cobb angle. Though standing imbalance was reported to be more significant in curves of 15° or more [12], no correlation with the spinal deformity progression has yet been reported. Stylianides et al. [3] suggest that an asymmetrical bone growth is part of the pelvic rotation-inducing system transferred to the spine to initiate scoliosis [15] and trunk distortion. This phenomenon could be responsible for the observed instability about the vertical axis [26]. Altered iliac spine growth in the transverse plane of non-treated adolescent idiopathic scoliosis girls was found to be correlated with Cobb angles [43]. This horizontal morphologic imbalance could be in part compensated by muscle action resulting in a greater free moment previously reported in girls with moderate and severe spinal deformity [28].

These results support in part the contentions expressed by Weiss et al. [39,40]. They advocate physiotherapy to avoid brace treatment or in case of a brace intensive rehabilitation programs when indicated [39]. But when brace treatment is the major issue, then physiotherapy is secondary [40]. Though exercise based therapies with or without orthopedic management was reported to be controversial [40], we suggest that exercises challenging balance and developing ankle proprioception to control the amplitude of the balance control commands [41] could prove beneficial.

It is unclear if standing imbalance is a consequence of the body posture asymmetry and pelvic distortion, or if it declines slowly, or if its onset takes place later on. Based on the pathogenesis concept proposed by Burwell et al. [15], we suggest that, for girls with a right thoracic scoliosis, pelvic abnormal growth modifies the relation between the center of mass of the whole body and that of the trunk as scoliosis progresses [26]. As the body center of mass moves backwards [10], the scoliotic girls stand more on their heels. This exacerbates the horizontal standing balance [4] and increases the demand on torsional control [28]. In more severe scoliosis, it appears that a combination of pelvic asymmetry and the deterioration of the neuromuscular control [13] leads to perturbed gait initiation [14] and gait itself [42].

## Conclusions

This study has shown that pelvic distortion, body posture asymmetry, and standing imbalance of scoliotic girls are more pronounced when compared to a comparable able-bodied group. Upright stance in able-bodied girls is mostly characterized by the control they exercise to maintain balance since there is little pelvic distortion and their posture is relatively erect. Untreated scoliotic girls were shown to have an abnormal pelvic morphology combined with body posture asymmetries. Their ability to maintain balance is also perturbed, but it could be a compensatory mechanism or one that develops or manifests itself in the later stage of the scoliotic progression.
